# Hungry Bone Syndrome After Parathyroidectomy for Secondary Hyperparathyroidism: Pathogenesis and Contemporary Clinical Considerations

**DOI:** 10.3390/jcm14197104

**Published:** 2025-10-09

**Authors:** Adina Coman, Cristi Tarta, Marco Marian, Daian Ionel Popa, Sorin Olariu, Mihai Rosu, Diana Utu, Florina Buleu, Anca-Monica Macovei-Oprescu, Dorin Novacescu, Flavia Zara, Marius Murariu

**Affiliations:** 1 Researching Future Surgery II Research Center, Department X, Discipline of General Surgery II, Faculty of Medicine, Victor Babes University of Medicine and Pharmacy Timisoara, E. Murgu Square, No. 2, 300041 Timisoara, Romania; adina.coman@umft.ro (A.C.); marian.marco@umft.ro (M.M.); 2 Doctoral School, Victor Babes University of Medicine and Pharmacy Timisoara, E. Murgu Square, No. 2, 300041 Timisoara, Romania; daian-ionel.popa@umft.ro; 3 Department X, Discipline of General Surgery I, Faculty of Medicine, Victor Babes University of Medicine and Pharmacy Timisoara, E. Murgu Square, No. 2, 300041 Timisoara, Romania; olariu.sorin@umft.ro (S.O.); murariu.marius@umft.ro (M.M.); 4 Department of Medicine, Discipline of Surgery I, Vasile Goldiş Western University, Liviu Rebreanu Boulevard, No. 86, 310414 Arad, Romania; rosu.mihai@uvvg.ro; 5 Department II, Discipline of Pharmacology-Pharmacotherapy, Faculty of Pharmacy, Victor Babes University of Medicine and Pharmacy Timisoara, E. Murgu Square, No. 2, 300041 Timisoara, Romania; diana.utu@umft.ro; 6 Department VI, Discipline of Internal Medicine and Ambulatory Care, Prevention and Cardiovascular Recovery, Faculty of Medicine, Victor Babes University of Medicine and Pharmacy Timisoara, E. Murgu Square, No. 2, 300041 Timisoara, Romania; florina.buleu@umft.ro; 7 Gastroenterology, Agrippa Ionescu Emergency Clinical Hospital, Bucharest, Romania, University of Medicine and Pharmacy Carol Davila Bucharest, Eroii Sanitari Bvd., no. 8, Sector 5, 300041 Bucharest, Romania; anca.macovei@umfcd.ro; 8 Department II of Microscopic Morphology, Victor Babes University of Medicine and Pharmacy Timisoara, E. Murgu Square, No. 2, 300041 Timisoara, Romania; flavia.zara@umft.ro

**Keywords:** hungry bone syndrome, secondary hyperparathyroidism, parathyroidectomy, chronic kidney disease, bone turnover markers, hypocalcemia, calcium-sensing receptor, RANKL/OPG pathway, Wnt/β-catenin signaling, endocrine surgery complications

## Abstract

Secondary hyperparathyroidism (SHPT) in chronic kidney disease often necessitates parathyroidectomy (PTX), but this definitive treatment can precipitate hungry bone syndrome (HBS)—a profound, prolonged hypocalcemia caused by the rapid skeletal uptake of minerals after surgery. HBS results from the abrupt cessation of parathyroid hormone (PTH)-driven bone resorption while bone formation continues, leading to intensive mineral deposition (mainly calcium) into chronically demineralized bone. Clinically, HBS ranges from asymptomatic biochemical disturbances to life-threatening hypocalcemia with tetany, seizures, and/or cardiac arrhythmias. This illustrative review synthesizes current knowledge of HBS pathogenesis and management in the context of SHPT. We detail how the high-turnover bone remodeling state of SHPT (osteitis fibrosa cystica) creates an expansive unmineralized osteoid pool that avidly mineralizes post-PTX. We also explore molecular mechanisms (e.g., RANKL/OPG dysregulation, Wnt/β-catenin activation, osteocyte-driven signals, and calcium-sensing receptor effects) that underpin this process. Key preoperative risk factors for HBS include very elevated PTH and alkaline phosphatase levels, large skeletal calcium deficits, younger patient age, and total PTX. We outline the typical postoperative course of HBS, phased from immediate acute hypocalcemia to a nadir and gradual recovery. Prevention and management strategies are emphasized, centered on vigilant monitoring and aggressive calcium and calcitriol supplementation, with preoperative optimization (e.g., vitamin D loading, calcimimetics) to mitigate severity. By enhancing risk stratification and perioperative care, clinicians can improve outcomes and safely navigate patients through this challenging complication of endocrine surgery.

## 1. Introduction

Secondary hyperparathyroidism (SHPT) represents one of the most challenging complications of chronic kidney disease (CKD), affecting virtually all patients with advanced renal insufficiency and contributing significantly to the morbidity and mortality burden in this population [1,2]. The pathophysiology of SHPT involves a complex interplay of mineral metabolism derangements, beginning early in CKD progression when glomerular filtration rate (GFR) remains relatively preserved, and culminating in severe skeletal and cardiovascular complications in end-stage renal disease (ESRD) [3,4].

Despite advances in medical management including vitamin D receptor activators, calcimimetics, and phosphate binders, ~15–40% of dialysis patients (proportion increasing with dialysis vintage) with severe SHPT will ultimately require parathyroidectomy (PTX) to control medication refractory disease and its associated complications [5,6]. However, contemporary practice increasingly reserves PTX for refractory SHPT after a trial of optimized medical therapy (e.g., calcimimetics, vitamin D analogs, and phosphate control), reflecting a real-world reduction or delay in surgical need for many patients. Even so, PTX remains indispensable when targets are not achieved or complications ensue (e.g., bone pain/fracture, calciphylaxis, intractable pruritus, or very high/escape PTH) [5].

The surgical management of SHPT, while highly effective in controlling parathyroid hormone (PTH) hypersecretion and improving clinical outcomes, carries the risk of precipitating hungry bone syndrome (HBS), a severe metabolic complication characterized by rapid, profound, and prolonged hypocalcemia following PTX [7,8]. First described in 1949 [9], HBS occurs when the sudden withdrawal of PTH leads to an abrupt cessation of osteoclast-mediated bone resorption, while osteoblastic bone formation continues unabated, creating a massive influx of calcium, phosphate, and magnesium into previously demineralized bone matrix [10]. This dramatic shift in mineral metabolism can result in life-threatening hypocalcemia, requiring intensive monitoring and aggressive supplementation for weeks to months postoperatively [11,12].

The incidence of HBS following PTX for SHPT varies widely in the literature, ranging from ~20–30% in older series to >80% in recent cohorts (when including milder cases), with this variability reflecting differences in diagnostic criteria, patient populations, and perioperative management protocols [13,14]. The clinical spectrum of HBS ranges from asymptomatic biochemical abnormalities to severe manifestations including tetany, seizures, laryngospasm, and cardiac arrhythmias due to QT prolongation [15,16]. Risk factors for developing severe HBS include markedly elevated preoperative PTH levels (>1000 pg/mL), high bone turnover markers (BTMs)—particularly alkaline phosphatase (ALP) >3–4×the upper limit of normal (ULN), younger age, larger body size, absence of preoperative hypercalcemia, and the extent of parathyroid tissue removal [17,18].

The pathogenesis of HBS in SHPT patients is fundamentally rooted in the high-turnover bone disease (osteitis fibrosa cystica) that develops during prolonged PTH excess [19]. Chronic PTH elevation drives excessive bone remodeling through sustained upregulation of the receptor activator of nuclear factor-κB ligand (RANKL)/osteoprotegerin (OPG) system, leading to increased osteoclast recruitment and activity [20]. Simultaneously, PTH suppresses sclerostin production by osteocytes, enhancing Wnt/β-catenin signaling and promoting osteoblast proliferation [21]. This creates an expanded pool of unmineralized osteoid matrix that, following PTX, starts to become rapidly mineralized (as the inhibitory effect of PTH is removed) [22]. The skeletal calcium deficit accumulated during years of hyperparathyroidism, estimated at ~15–20% of total body calcium in severe cases, drives the voracious uptake of minerals that characterizes HBS [23].

Recent advances in our understanding of bone biology have revealed additional molecular mechanisms contributing to HBS pathophysiology. The calcium-sensing receptor (CaSR) expressed on bone cells may amplify the skeletal response to hypocalcemia following PTX, further stimulating mineral uptake [24]. Fibroblast growth factor-23 (FGF-23) and its co-receptor Klotho, both markedly dysregulated in CKD, influence post-PTX mineral dynamics, through their effects on vitamin D metabolism and phosphate handling [25]. Genetic polymorphisms in vitamin D receptor (VDR), CaSR, and components of bone remodeling pathways may modulate individual susceptibility to HBS, though these associations require further validation [26].

The management of HBS remains largely supportive, focusing on aggressive calcium and vitamin D supplementation to meet the enormous skeletal demand, while maintaining safe serum calcium levels [27]. Prophylactic strategies, including preoperative vitamin D loading, bisphosphonate therapy, and calcimimetic optimization, have shown variable efficacy [28]. Novel approaches targeting specific molecular pathways, such as anti-RANKL antibodies or sclerostin inhibitors, remain investigational [29]. The optimal surgical approach—total versus subtotal PTX with or without autotransplantation—continues to be debated, balancing the risk of HBS against the likelihood of persistent or recurrent hyperparathyroidism [30].

The current narrative literature review was informed by a meticulous PubMed/MEDLINE database search (latest update August 2025) for English-language articles on “hungry bone syndrome,” “secondary hyperparathyroidism,” “parathyroidectomy,” and related keywords. Relevant original studies, reviews, and clinical guidelines were identified and screened. Priority was given to contemporary evidence (including large cohort studies and recent systematic reviews) and seminal earlier works to ensure both up-to-date and foundational perspectives. We synthesized the reference information qualitatively to highlight areas of consensus as well as conflicting evidence, and to provide an integrated overview of HBS pathogenesis and management.

All in all, this comprehensive review aims to synthesize current knowledge regarding the pathogenesis, risk factors, clinical evolution, and the management of HBS following PTX for SHPT in CKD patients. We integrate insights from nephrology, endocrinology, and surgery to provide a contemporary framework for understanding this challenging complication. By examining the molecular mechanisms underlying the transition from high-turnover bone disease to the “hungry bone” state, we aim to inform evidence-based approaches to risk stratification, prevention, and treatment. Furthermore, we highlight emerging concepts and future directions that may lead to improved outcomes for the growing population of CKD patients requiring surgical management of SHPT.

## 2. Parathyroid Hormone Physiology

PTH is a pivotal regulator of calcium and phosphate homeostasis, orchestrating complex molecular mechanisms, crucial for maintaining mineral balance and bone integrity [31]. It is initially synthesized as a pre-hormone (pre-pro-PTH), cleaved to an 84-amino-acid peptide, (1–84)PTH or intact (i)PTH, and stored in secretory granules awaiting either circadian and pulsatile release, or intracellular degradation, within the chief cells of the parathyroid glands [32]. This secretion is finely regulated by extracellular calcium via the CaSR: hypocalcemia triggers a surge of biologically active iPTH release, whereas hypercalcemia suppresses overall PTH secretion and shifts it towards the preferential production of less active C-terminal PTH fragments [33].

Once in circulation, PTH undergoes rapid metabolization, primarily in the liver and kidneys, generating various fragments with heterogeneous bioactivity and half-lives [34]. The iPTH molecule has a short half-life of ~2–4 min, while its C-terminal fragments, i.e., (7–84)PTH, persist longer and may account for ~50% of circulating PTH [35]. Circulating PTH represents a mixture of intact and fragmented peptides that exert distinct biological effects through differential interactions with PTH receptors and modulation of receptor sensitivity [36]. The kidneys normally clear these PTH fragments; thus, advanced renal failure causes accumulation of C-terminal PTH species [37]. A receptor specific to the C-terminal region of PTH (C-PTHR) has been recently identified, primarily in osteocytes. Even though its physiological relevance remains under investigation, it appears to act antagonistically to the PTH/PTH1R pathway, potentially suppressing osteocytic osteolysis and modulating the skeletal response to PTH [38].

Conversely, the main biological implications of PTH are predominantly mediated through its binding to the type 1 PTH receptor (PTH1R), a G protein-coupled receptor highly expressed on osteoblasts, osteocytes, and renal tubular cells [39]. PTH1R activation stimulates intracellular protein kinase A (PKA) and C (PKC) signaling cascades that alter gene expression involved in bone remodeling and mineral transport [40]. In bone, PTH1R signaling in osteoblast-lineage cells has dual effects—directly stimulating osteoblastic bone formation, while indirectly stimulating osteoclastic bone resorption through osteoblast-derived signaling pathways [41].

Beyond skeletal targets, PTH acts on the kidneys to enhance calcium reabsorption in the distal tubule and collecting duct through upregulation of transient receptor potential vanilloid 5 (TRPV5) channels and calbindin-D28k [42]. Simultaneously, PTH inhibits phosphate reabsorption in the proximal tubule by promoting internalization and degradation of sodium-phosphate co-transporters (NaPi-2a and NaPi-2c) [43]. PTH also stimulates 1α-hydroxylase (CYP27B1) activity in proximal tubular cells, enhancing conversion of 25-hydroxyvitamin D to active calcitriol while suppressing 24-hydroxylase (CYP24A1), the enzyme responsible for vitamin D catabolism [44]. The resulting rise in calcitriol augments intestinal calcium absorption through upregulation of TRPV6 channels and provides negative feedback to the parathyroid glands via the VDR. Additional non-classical targets include pancreas [45], bone marrow [46] and vascular smooth muscle cells (influencing vascular tone through acute vasodilatory effects and reduction in vascular oxidative stress) [47], reflecting PTH’s systemic role, beyond mineral metabolism modulation.

Understanding these normal physiological mechanisms of PTH action and metabolism provides a foundation for recognizing the pathological alterations that arise in conditions like SHPT and HBS, further discussed subsequently.

## 3. Pathogenesis of Secondary Hyperparathyroidism in Chronic Kidney Disease

Under normal physiological conditions, PTH exerts crucial regulatory effects on renal calcium and phosphate handling to maintain mineral homeostasis. As noted above, the hormone enhances calcium reabsorption (in the distal tubule and collecting duct), while promoting phosphate excretion (through downregulation of sodium-phosphate co-transporters in the proximal tubule) [42,43]. PTH also stimulates 1α-hydroxylase activity in proximal tubular cells, enhancing the conversion of 25-hydroxyvitamin D to active calcitriol (1,25-dihydroxyvitamin D), which in turn augments dietary calcium absorption [44]. These coordinated actions help maintain calcium-phosphate balance in health.

CKD profoundly disrupts calcium-phosphate homeostasis through multiple interconnected mechanisms, leading to SHPT as an initially adaptive but ultimately maladaptive response [48]. This condition is characterized by parathyroid hyperplasia and persistent PTH overproduction in response to hypocalcemia, hyperphosphatemia, and decreased calcitriol synthesis [49]. In fact, emerging evidence from recent large cohort studies indicates that PTH elevation begins early on in CKD, even when estimated glomerular filtration rate (eGFR) is well above 45 mL/min/1.73 m^2^ (the traditional threshold [50]), with measurable significant increases observed at eGFRs as high as 126 mL/min/1.73 m^2^ [51]. Onward, as eGFR further declines, PTH levels increase proportionally, eventually reaching 10–100× the ULN in dialysis patients [52]. This state of overproduction results in a high bone turnover phenotype, driven by the continuous stimulation of osteoclasts and osteoblasts via elevated PTH levels.

SHPT development in CKD is multifactorial, yet follows a predictable pathophysiological cascade. As kidney function declines, phosphate retention occurs first, triggering a cascade of hormonal adaptations [53]. Elevated phosphate levels stimulate FGF-23 production, which suppresses 1α-hydroxylase activity, leading to decreased calcitriol synthesis [54]. The resulting calcitriol deficiency impairs intestinal calcium absorption and reduces negative feedback on the parathyroid glands [54]. Simultaneously, hyperphosphatemia directly stimulates PTH secretion and promotes parathyroid cell proliferation [53]. Normal inhibitory feedback mechanisms become deficient due to downregulation of the parathyroid CaSR, VDR, and FGF-23/Klotho receptors [55]. Thus, phosphate retention, elevated FGF-23 levels and decreased calcitriol synthesis, act synergistically with the subsequent calcium depletion to stimulate PTH hypersecretion. Meanwhile, receptor downregulation and end-organ hyporesponsiveness exacerbate the need for progressively higher PTH levels to maintain mineral balance [37,53,54,55,56].

Furthermore, CKD also fundamentally alters PTH metabolism. The kidney’s impaired ability to clear PTH fragments results in accumulation of C-terminal PTH species, particularly (7–84)PTH fragments [37]. These fragments may act on C-PTHR or competitively inhibit PTH1R signaling, thereby antagonizing the biological effects of full-length (1–84)PTH [36,37,38]. Clinical data show a proportional increase in C-terminal PTH fragments versus iPTH during CKD progression [36,37,38]. Simultaneously, posttranslational oxidative modifications, particularly methionine oxidation at positions 8 and 18, will additionally diminish the bioactivity of iPTH, further contributing to end-organ resistance [57]. Oxidation of methionine residue 8 seems especially crucial in generating conformational changes in the secondary structure of PTH [57,58].

Finally, PTH receptor signaling becomes progressively impaired in CKD. Experimental CKD models demonstrate decreased PTH1R expression in both bone and renal tissue, with receptor mRNA levels reduced by ~50–70% in severe uremia [59]. This downregulation most likely occurs as a result of oxidative stress, inflammation/acidosis, uremic toxins (such as indoxyl sulfate), inflammatory bioactive lipids (e.g., ox-LDL), and/or the excessive presence of C-terminal PTH fragments [60]. Soluble Klotho, a co-receptor induced by FGF-23, may further interfere with PTH1R signaling by directly interacting with the receptor on renal tubular cells and preventing bioactive iPTH binding [47], while high levels of sclerostin and OPG may blunt downstream anabolic signaling pathways [61]. In fact, circulating OPG and sclerostin levels are many times higher in advanced CKD than in individuals with normal kidney function [61]. Therefore, this combination of receptor downregulation, accumulation of iPTH-antagonistic C-terminal fragments, and uremic toxin interference creates a state of increasingly important PTH resistance.

## 4. Molecular Targets and Signaling Pathways in Bone Tissue

**Figure 1:**
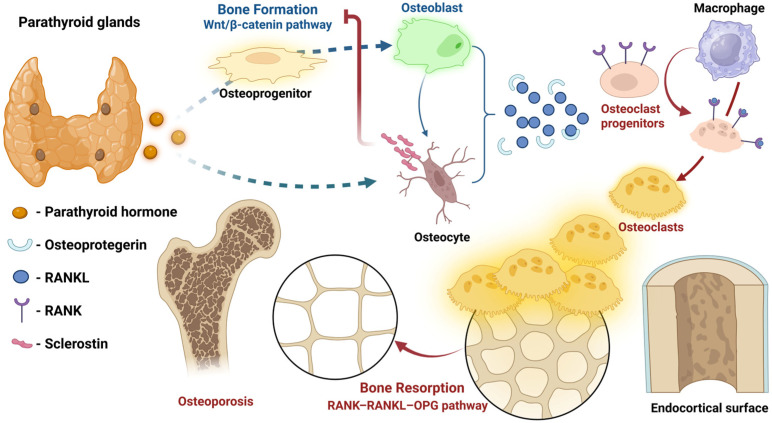
**Parathyroid hormone (PTH)-mediated bone metabolism.** PTH binding to type 1 receptors on osteoblasts/osteocytes activates Wnt/β-catenin signaling, accelerating osteoblastic mesenchymal differentiation. Concurrent receptor activator of nuclear factor-κB ligand (RANKL) upregulation stimulates osteoclastogenesis, while osteoprotegerin acts as RANKL decoy receptor. PTH suppresses sclerostin, enhancing Wnt signaling. End result: balanced, tightly regulated, bone formation and resorption. Illustration created using BioRender [64].

Within bone, PTH exerts dual anabolic and catabolic effects, dependent on exposure patterns [41], as seen in Figure 1. Intermittent, pulsatile exposure predominantly activates anabolic pathways, both directly, and indirectly, through the Wnt/β-catenin signaling cascade, leading to osteoblast differentiation and maturation [41]. In this context, PTH also represses the osteocyte-derived Wnt signaling antagonist sclerostin, thereby further promoting osteogenesis and increasing bone mass [41]. Conversely, sustained PTH elevation (as occurs in SHPT) activates catabolic processes, primarily through the receptor activator of the RANKL/RANK/OPG system [41,62]. Chronic PTH excess drives osteoblasts and osteocytes to secrete RANKL, the key stimulator of osteoclastogenesis, while reducing the release of its decoy receptor OPG and causing sustained upregulation of monocyte chemoattractant protein-1 (MCP-1—another osteolytic mediator) [63]. The result is an uncoupling of the remodeling cycle, tipping the balance towards osteoclast differentiation and activation, enhancing bone resorption and mobilizing calcium and phosphate into circulation, i.e., ultimately determining osteoporosis, if left unchecked (see Figure 1).

**Figure 2:**
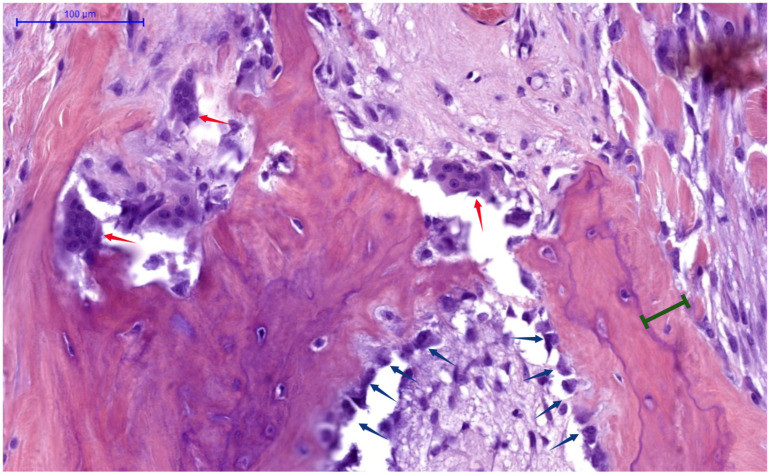
**Histological human bone section (H&E stain) demonstrating high-turnover remodeling**. Multinucleated osteoclasts (red arrows) in resorption lacunae, are actively breaking down bone matrix, while osteoblasts (blue arrows), lining trabecular surfaces, are depositing osteoid seams (green bracket). NB: Archival image from Histology slide bank, processed in Biorender [64].

Figure 2 provides histological evidence of these cellular activities, demonstrating active human bone remodeling sites, with the following pathognomonic elements: osteoclasts (indicated by red arrows) actively resorbing bone matrix; osteoblasts (blue arrows) laying down new osteoid; and osteoid seams (green interval lines), which represent newly deposited bone matrix, awaiting mineralization.

### 4.1. The RANKL/RANK/OPG System

The RANKL/RANK/OPG system serves as the primary regulator of osteoclastogenesis and bone resorption (see Figure 1) [62]. RANKL is a member of the tumor necrosis factor superfamily expressed by osteoblasts, osteocytes, and activated T cells that binds to its receptor RANK on osteoclast precursors, inducing their differentiation and functional activation [62,63]. OPG, produced by osteoblast-lineage cells, acts as a soluble decoy receptor that binds RANKL and prevents its interaction with RANK, thereby inhibiting osteoclastogenesis [65]. Under normal conditions, this system maintains skeletal homeostasis by balancing bone resorption versus osteogenesis. However, chronic PTH elevation fundamentally disrupts this equilibrium [20].

In osteoblasts/osteocytes, sustained PTH over-secretion (i.e., PTH1R signaling) leads to increased RANKL expression, while simultaneously suppressing OPG production [65]. This shift in the RANKL/OPG ratio strongly favors osteoclastogenesis, driving excessive differentiation of monocyte–macrophage precursors into mature, multinucleated osteoclasts (see Figure 1), which then create numerous deep resorption lacunae in bone tissue and intensively release calcium/phosphate from the skeletal matrix. This acceleration of bone resorption, characterizes the high-turnover bone disease and skeletal fragility seen in SHPT [66]. Importantly, this process is accompanied by reactive increases in osteoblastic activity, though the coupling between resorption and osteogenesis becomes increasingly dysregulated as HPT progresses [67].

Following PTX, the sudden withdrawal of PTH triggers an immediate and dramatic reversal of these signaling dynamics. Within hours of surgery, RANKL expression begins to fall dramatically, while OPG levels start to recover, effectively halting osteoclastic differentiation and activity [68]. Molecular analyses of bone biopsies have demonstrated that RANKL mRNA expression decreases by 60–80% within 48 h post-PTX, while OPG expression increases by 200–300% [69,70,71]. This molecular switch creates a powerfully anti-resorptive environment, where existing osteoclasts undergo accelerated apoptosis and new osteoclast formation ceases.

However, the osteoblastic response follows a markedly different trajectory. Osteoblasts that were activated during the hyperparathyroid state continue their bone-forming activities unabated, for days to weeks after PTH withdrawal [72]. This persistence is partly explained by the longer lifespan of osteoblasts (~3 months) compared to osteoclasts (2–3 weeks), and by the continued presence of pro-osteogenetic signals, including insulin-like growth factor-1 (IGF-1), bone morphogenetic proteins (BMPs), and transforming growth factor-β (TGF-β), that were released from the bone matrix during the preceding period of accelerated resorption [73,74]. The temporal uncoupling between rapidly suppressed resorption and persistently elevated bone formation creates the fundamental pathophysiological substrate for HBS.

### 4.2. The Wnt/β-Catenin Signaling Pathway

The Wnt/β-catenin pathway governs the regulation of osteoblast differentiation, survival, and function, and is thus critically involved in the pathogenesis of HBS [75]. Canonically, Wnt proteins bind to Frizzled receptors and LRP5/6 co-receptors on osteoblast-lineage cells, stabilizing β-catenin and promoting its translocation into the nucleus, where it drives the transcription of pro-osteogenic genes (i.e., *RUNX2*, *Osterix*, and type I collagen) [75]. Conversely, this pathway is tightly regulated by inhibitory molecules such as sclerostin (encoded by *SOST*) and Dickkopf-1 (*DKK-1*), which prevent Wnt receptor activation [76,77].

In a quiescent state, osteocytes produce sclerostin, which inhibits Wnt signaling and thus keeps bone formation in check. PTH acutely downregulates sclerostin production, through PKA-mediated phosphorylation of cAMP-response element binding protein (CREB) and Myocyte Enhancer Factor 2 (MEF2), thereby enhancing Wnt signaling and promoting bone formation [78,79]. With chronic PTH excess, continuous sclerostin suppression leads to dysregulated Wnt signaling, contributing to the high osteoblastic activity seen in SHPT, yet the simultaneous upregulation of RANKL will dominate, resulting in ongoing net bone loss. Herein, immunohistochemistry studies of bone biopsies from SHPT patients have demonstrated markedly reduced sclerostin expression in osteocytes, with levels inversely correlating with serum PTH and bone formation rates [80]. Paradoxically, increased osteocytic sclerostin expression has been observed in early-stage CKD, suggesting complex stage-dependent regulation [81]. Following PTX, sclerostin expression recovers gradually over several weeks. During this transition, Wnt signaling remains elevated, contributing to the continued osteoblastic activity characterizing HBS.

Lastly, beyond sclerostin production, osteocytes, comprising 90–95% of all bone cells, function as mechanosensors and orchestrators of bone remodeling [82]. Through their extensive lacunar-canalicular network, they communicate with surface osteoblasts and osteoclasts, coordinating remodeling responses [83]. PTH-mediated calcium release encompasses both rapid skeletal exchangeable pools and osteocytic osteolysis (i.e., resorption of bone matrix by osteocytes) for acute responses (minutes to hours), whereas slower responses, over days, involve bone remodeling cells. Chronic PTH elevation alters osteocyte function, increasing production of RANKL and other regulatory factors [84]. Sudden PTH depletion after PTX disrupts this established signaling network, contributing to the dysregulated remodeling underlying HBS.

### 4.3. Calcium-Sensing and Mineral Flux Dynamics

The CaSR plays a crucial but underappreciated role in HBS pathophysiology. Bone cells themselves (i.e., osteoblasts and osteoclasts) can directly sense extracellular calcium concentrations through CaSR expressed on their surface [85]. This local calcium-sensing mechanism plays a supportive role in bone turnover regulation, which becomes particularly relevant during the rapid metabolic shifts in HBS. In chronic HPT, persistent hypercalcemia may lead to CaSR desensitization, requiring higher calcium levels to suppress PTH secretion and modulate bone cell function [86]. Following PTX, rapid calcium decline creates a strong stimulus for bone formation through CaSR-mediated pathways, potentially exacerbating the “hungry bone” phenomenon [87].

The magnitude of calcium flux into bone during HBS is remarkable. Severe HBS can involve net calcium accretion rates of 1000–2000 mg/day, far exceeding the normal dietary calcium absorption capacity of 200–400 mg/day [88]. This creates an insurmountable gap between calcium supply and demand, explaining the profound HBS hypocalcemia despite aggressive supplementation. The skeleton’s calcium deficit accumulated during years of HPT—~20% of total skeletal calcium content in severe cases—drives this avid mineral uptake [89].

Phosphate dynamics add complexity. Despite absent kidney function in dialysis patients, serum phosphate often decreases dramatically after PTX, sometimes <2.0 mg/dL [30]. This occurs because phosphate incorporates into newly forming hydroxyapatite crystals alongside calcium at a Ca^2+^:PO_4_^3−^ ratio of approximately 1.67:1 [90]. PTH removal also eliminates its phosphaturic effect on any residual renal function and reduces phosphate release from bone [91]. Hypomagnesemia frequently accompanies HBS, as magnesium is incorporated into bone mineral and serves as a cofactor for numerous bone formation enzymes [27].

## 5. Bone Remodeling Dynamics Before and After Parathyroidectomy

Understanding normal bone remodeling provides essential context for comprehending HBS pathophysiology. The bone remodeling cycle occurs in discrete anatomical structures called basic multicellular units (BMUs), which consist of teams of osteoclasts and osteoblasts working in coordinated sequence [92]. In healthy adults, ~1 million BMUs are active at any time, with each cycle lasting 3–6 months and proceeding through five phases: activation, resorption, reversal, formation, and termination [93]. Tight coupling ensures that the amount of bone formed equals that resorbed, maintaining skeletal integrity.

In SHPT, chronic PTH elevation profoundly disrupts this orderly process. The activation frequency of new BMUs increases dramatically, from the normal 0.1–0.2/year to as high as 2–3/year in severe cases [94]. The resorption phase becomes accelerated and exaggerated, with osteoclasts creating abnormally deep resorption cavities (see Figure 2). Histomorphometric analysis reveals classic features of osteodystrophy: increased osteoclast numbers (often 10–20× above normal), extensive resorption surfaces (>15% compared to normal <2%), peritrabecular fibrosis, and woven bone formation—unmineralized osteoid seams [95], as seen in Figure 2. Additionally, marrow fibrosis and formation of brown tumors occur in severe cases. Despite compensatory increases in osteoblast numbers, the rate of bone formation cannot match the accelerated resorption, leading progressively expanding osteoid volume, net bone loss and architectural deterioration [96].

The pre-PTX skeleton thus represents a battlefield of accelerated and chaotic remodeling. Multiple BMUs in various stages of the remodeling cycle create a heterogeneous landscape of resorption cavities, unmineralized osteoid, and partially mineralized new bone [97]. Importantly, the total osteoid volume—representing unmineralized bone matrix awaiting mineral deposition—can increase 5–10× in severe SHPT [98]. This expanded osteoid compartment becomes critically important after PTX, as it represents a massive potential “metabolic sponge” for calcium, phosphate and magnesium, once mineralization is no longer inhibited by high PTH levels. Notably, hypomagnesemia, common in dialysis patients, impairs both PTH secretion and receptor responsiveness, compounding hypocalcemia [99].

**Figure 3:**
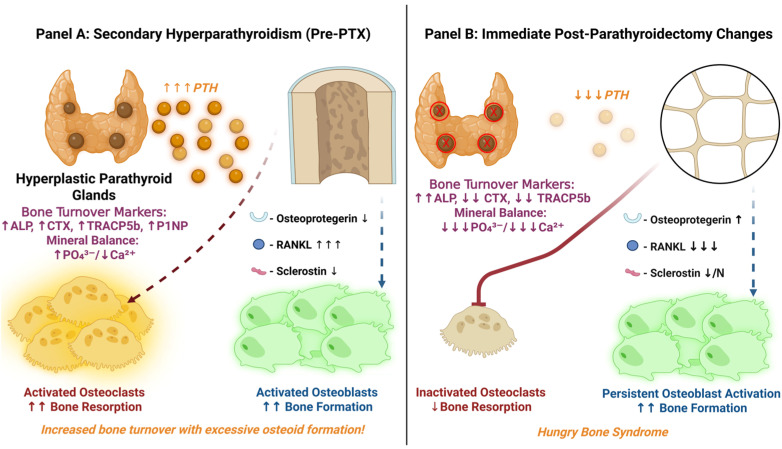
**Hungry Bone Syndrome (HBS) Pathophysiology:** (**A**) **Before parathyroidectomy (PTX)**—hyperplastic parathyroids secrete excess parathyroid hormone (PTH), driven by CKD-induced hypocalcemia, hyperphosphatemia, and reduced active vitamin D. High PTH increases receptor activator of nuclear factor-κB ligand (RANKL)/decreases osteoprotegerin (OPG) expression and suppresses sclerostin, causing high bone resorption and turnover, with excessive unmineralized osteoid (osteodystrophy) and elevated bone turnover markers, i.e., alkaline phosphatase (ALP), tartrate-resistant acid phosphatase 5b (TRACP5b), C-terminal telopeptide (CTX), and procollagen type I N-terminal propeptide (P1NP). (**B**) **Immediate post-PTX changes**—the abrupt PTH drop rapidly inhibits osteoclast-mediated bone resorption (diminished RANKL, with increased OPG), while osteoblast activity initially continues unhindered (sclerostin production normalizes slowly), prompting the rapid uptake of calcium (Ca^2+^) and phosphate (PO_4_^3−^) into previously unmineralized bone matrix. This sudden mineral shift markedly reduces circulating serum Ca^2+^ and PO_4_^3−^, manifesting clinically as HBS. Serum biomarkers dynamically respond, showing drastically decreased PTH, CTX, and TRACP5b, with transient elevation in ALP as mineralization intensifies. Illustration created using BioRender [64].

Serum biomarkers reflect these dramatic changes in bone metabolism. In severe SHPT, iPTH levels can reach 10–100× the ULN [52,100,101]. Bone formation markers like ALP, particularly the bone-specific isoenzyme (BALP) [102], osteocalcin (OC) [103], and procollagen type I N-terminal propeptide (P1NP) [104] are markedly elevated, indicating active osteoblasts and matrix synthesis. These osteogenesis markers have shown consistent 2–5× elevations, with specific isoforms unique to uremic patients. Concurrently, bone resorption markers such as tartrate-resistant acid phosphatase 5b (TRACP5b) [105], C-terminal telopeptide (CTX) and N-terminal telopeptide (NTX) [106] are increased, often reaching the upper limits of assay detection (see Figure 3). In dialysis patients with severe SHPT, pre-PTX levels of TRACP5b were 3–4× over the ULN [105]. Conversely, while extreme CTX/NTX elevations (20-fold) are reported in clinical literature, large cohort studies document more moderate 3–5× increases [107], suggesting the highest values occur in end-stage cases typically reported in smaller surgical series.

Figure 3 illustrates the comparative bone turnover dynamics seen perioperatively during SHPT management, i.e., before and after PTX. Figure 3A depicts the pre-PTX high-turnover state of SHPT (excess PTH stimulates osteoclast-mediated bone resorption and excessive osteoid formation, with elevated BTMs—ALP, CTX, TRACP5b and P1NP—overall, and the typical biochemical pattern of high phosphate and low calcium).

**Figure 4:**
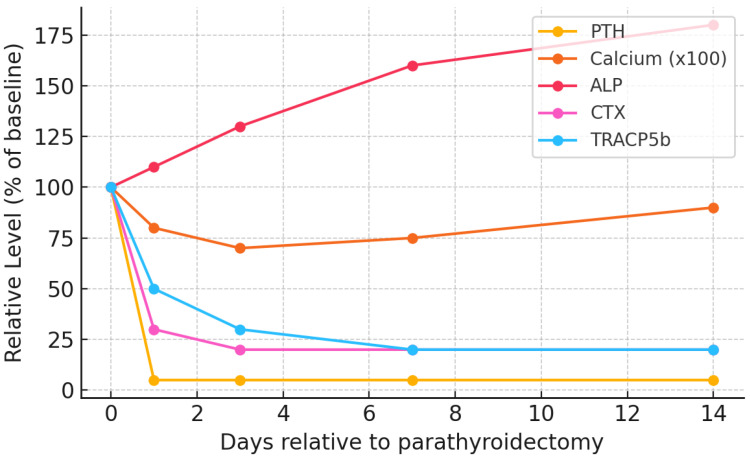
**Schematic summary of postoperative trajectories for key biomarkers in hungry bone syndrome.** Post-parathyroidectomy (day 0), serum PTH levels fall precipitously, removing the stimulus for bone resorption [27]. Osteoclast activity markers (e.g., CTX, TRACP5b) decline accordingly [109,110]. In contrast, osteoblast activity remains elevated or even increases, as previously expanded osteoid is mineralized—reflected by a rise in alkaline phosphatase (ALP) in the following days/weeks post-op [30]. This uncoupling leads to a net flux of calcium from circulation into bone, causing serum calcium to drop to nadir within ~2–5 days post-op [30,111], before recovering with supplementation. Trends are idealized; calcium scale is expanded for visibility.

Conversely, in the immediate postoperative period (Figure 3B), especially when performing total PTX, within a few hours, iPTH levels plummet (from thousands of pg/mL to virtually zero). This abrupt withdrawal rapidly suppresses RANKL-mediated osteoclast activity—bone resorption is therefore rapidly attenuated. Consequently, bone resorption markers drop significantly within the first days post-PTX [108]: serum CTX typically falls by ~70% and TRACP5b by ~40% by day 3 [109,110] (as seen in Figure 4).

However, osteoblasts are initially unhindered by the post-PTX drop in PTH and remain transiently active. As mentioned previously, this uncouples bone formation from resorption, as these cells proceed to mineralize the previously expanded osteoid matrix. As calcium and phosphate are avidly incorporated herein, their circulating levels fall precipitously, with serum calcium reaching a nadir typically within 2–5 days post-op [30,111] (as shown in Figure 4). Phosphate levels also drop markedly, i.e., 2.28 ± 0.65 mmol/L to 1.75 ± 0.54 mmol/L on postoperative day 1 [30]. Magnesium similarly shifts into bone, and hypomagnesemia may require replacement therapy [112,113]. Lastly, this metabolic shift is reflected by a transient rise in ALP in the following days to weeks post-PTX [30].

In essence, the bones become “hungry” for calcium—hence the name HBS. The extent and severity of HBS are strongly correlated with the degree of preoperative bone turnover and the extent of PTH removal [17,27,114,115]. Total PTX without autotransplantation produces the most profound biochemical shifts and thus associates highest risk for severe hypocalcemia, whereas subtotal PTX or autotransplantation preserves residual parathyroid function, mitigating the severity of mineral imbalances [116]. Post-op management protocols, therefore, focus intensively on mineral supplementation (calcium, phosphate, magnesium) and active vitamin D analogs to counterbalance these rapid perioperative shifts and maintain electrolyte homeostasis until bone remodeling stabilizes [27].

To sum, HBS represents a dramatic remodeling shift following PTX: an abrupt cessation of bone resorption with sustained bone formation, triggering massive mineral uptake and symptomatic hypocalcemia. Individual susceptibility to HBS varies with SHPT severity, duration, pre-existing bone disease, nutritional status and surgical technique [18,30,117]. Emerging evidence suggests that genetic polymorphisms in the VDR, CaSR and components of the RANK-L/OPG and Wnt pathways may modulate bone turnover and hypocalcemia severity [118,119,120]. Recognizing the high-turnover bone state preop and anticipating this shift is critical in managing patients undergoing PTX for SHPT.

## 6. Timeline and Clinical Evolution

Clinically, the temporal evolution of HBS (as summarized in Table 1) follows a predictable pattern that reflects the underlying pathophysiological processes described above. Understanding this timeline is crucial for appropriate monitoring and management of patients undergoing PTX for SHPT.

The immediate postoperative period (0–6 h) is characterized by the precipitous fall in PTH levels, typically from pre-op values of 1000–3000 pg/mL to less than 50 pg/mL within 30 min of gland removal [121]. This rapid change initiates the aforementioned cascade of molecular events in bone remodeling, though clinical manifestations typically lag by 12–24 h due to the body’s calcium buffering capacity [122]. Thus, during this period, ionized calcium often remains relatively stable as extracellular calcium stores and calcium bound to albumin provide a temporary buffer. However, the cessation of PTH-mediated calcium reabsorption in the kidney (in patients with residual renal function) and the onset of bone resorption suppression set the stage for subsequent hypocalcemia.

The acute phase (12–72 h) witnesses the onset of more impactful biochemical changes and early clinical manifestations. Serum calcium begins declining within 18–24 h, with the rate of decline correlating strongly with pre-op bone turnover severity [123]. Initial symptoms are often subtle, including perioral tingling, digital paresthesias, and muscle cramps. Physical examination may reveal positive Chvostek’s sign (facial twitching when tapping the facial nerve) or Trousseau’s sign (carpopedal spasm with blood pressure cuff inflation), though these have limited sensitivity and specificity for predicting severe hypocalcemia. Serial calcium monitoring during this period is critical, as the rate of decline often accelerates between 24 and 48 h post-op [12]. Phosphate levels begin falling concurrently, though the magnitude is initially less dramatic [12,27]. Magnesium levels should also be monitored, as hypomagnesemia can develop early and exacerbate hypocalcemia [14].

The subacute phase (3–14 days) represents the period of maximal mineral imbalance and highest clinical risk. Serum calcium typically reaches its nadir around days 5–7, though this can vary from day 3 to day 14 depending on the severity of pre-existing bone disease [30]. Importantly, this timing coincides with peak ALP levels, reflecting maximal osteoblastic activity and bone formation [14]. In severe HBS cases, ionized calcium may fall below 0.8 mmol/L [30] (normal range: 1.15–1.30 mmol/L); for instance, one case, albeit of parathyroid carcinoma, reported post-PTX ionized calcium as low as 0.04 mmol/L despite aggressive supplementation [124]. Without appropriate management, severe manifestations may develop, including frank tetany, generalized seizures, bronchospasm, laryngospasm, and life-threatening cardiac arrhythmias [27]. Thus, heart failure can be precipitated or exacerbated due to impaired myocardial contractility [125]. Notably, the risk of sudden cardiac death from QT prolongation (proportional to the degree of hypocalcemia) and ventricular arrhythmias (torsades de pointes) is highest during this phase, mandating intensive monitoring and aggressive replacement therapy [126]. Conversely, generalized tonic–clonic seizures may further ensue with severe hypocalcemia, as well as neuropsychiatric symptoms including confusion, hallucinations, and depression [127].

The recovery phase extends from 2 weeks to 12 months or longer in severe cases. Gradual improvement in calcium levels occurs as bone formation rates normalize and the accumulated osteoid becomes fully mineralized [12]. ALP levels progressively decline, following a trajectory that can predict the duration of calcium supplementation requirements [123,128]. Most patients achieve biochemical stability by 3–6 months, though requirements for calcium and vitamin D supplementation show wide individual variation. Approximately 10–15% of patients experience “protracted HBS,” requiring more than one year for complete normalization, highlighting the potential for long-term metabolic consequences [16].

## 7. Risk Factors and Predictive Modeling for Hungry Bone Syndrome in Secondary Hyperparathyroidism

Not all patients with SHPT develop HBS after PTX, with reported incidence ranging from ~15% to over 80% in dialysis cohorts (i.e., variability reflecting differences in patient populations and HBS definitions) [13,14]. In fact, clinically there is a spectrum: from mild, transient hypocalcemia to severe, protracted HBS. Identifying those at risk is therefore critical.

### 7.1. Key Biochemical and Clinical Predictors

To date, a number of clinical, biochemical, and molecular risk factors have been identified in retrospective studies (summarized in Table 2).

Broadly, these predictors correspond to the severity of preexisting high-turnover bone disease and the abruptness of PTH withdrawal:

- **Preoperative PTH Level**: Very high iPTH represents a surrogate marker for severe SHPT and high bone turnover. Many studies report significantly higher pre-PTX PTH in HBS patients versus non-HBS [11]. In general, the risk of HBS rises sharply at the extreme PTH levels seen in dialysis patients (e.g., an iPTH >1000 pg/mL was reported as an independent HBS predictor in a 130-patient cohort [18]). However, some cohorts (see Table 2) did not find PTH an independent risk factor when controlling for ALP [30], likely because PTH and ALP are collinear.
- **Bone Turnover Markers**: Elevated serum ALP (especially bone-specific ALP) is one of the strongest and most consistent predictors of HBS. In multiple studies, pre-op ALP was significantly higher in those who developed HBS [11,18,30,114,129]. ALP reflects osteoblastic activity and overall bone turnover; values >3–4× the ULN (previously proposed cutoff of ALP >420 U/L [18]) carry high associated risk. Other predictive bone formation markers (OC, P1NP) and resorption markers (CTX, TRAP-5b) are currently being investigated and, when available, may prove useful [11,114]. Essentially, their dynamics reflect a very high bone turnover state pre-PTX, which sets the stage for dramatic post-op remineralization.
- **Preoperative Calcium and Vitamin D**: Paradoxically, lower pre-op serum calcium level (within the context of ESRD) portends a greater drop post-PTX. Patients with autonomous hypercalcemia from tertiary HPT (or adynamic bone) actually have less bone uptake capacity and thus lower HBS risk. Conversely, a normal or low calcium in a severe SHPT patient indicates suppressed bone mineralization despite high turnover—the body maintains normocalcemia by inhibiting calcium incorporation into an expanded but under-mineralized osteoid matrix. After PTX, this “hungry” skeleton rapidly mineralizes, causing profound hypocalcemia as calcium floods into bone. Indeed, absence of pre-op hypercalcemia was a significant risk factor in multiple studies, with meta-analyses showing an odds ratio of 0.19 (95% CI: 0.11–0.31) for severe post-op hypocalcemia [129]. Severe 25-hydroxyvitamin D deficiency (common in CKD) could theoretically exacerbate HBS by limiting baseline calcium stores, but most patients are repleted before PTX; studies on vitamin D status and HBS risk have shown mixed results [11].
- **Patient Factors (Age, Body Mass, Sex, Dialysis Vintage)**: Younger patients tend to mount more robust osteoblastic responses and have more metabolically active bone, which increases HBS susceptibility [11,18]. Indeed, age ≤45 was an independent predictor in multiple series [18,30]. Higher body weight (and by extension, greater skeletal mass) has also been linked to HBS risk [30]. The influence of sex is less clear. Some reports suggest males are at higher risk (potentially due to larger bone mass and lower estrogen levels predisposing to high-turnover lesions), but this has not been consistently observed. Nutritional status may play a role as well—one recent systematic review found that low pre-PTX albumin correlates with HBS [11], possibly reflecting frailty or chronic inflammation. Finally, the duration of pre-PTX dialysis may influence HBS risk, as longer dialysis vintage is associated with more severe CKD-mineral bone disorder. Multiple investigations have corroborated this notion [11,116].
- **Skeletal Burden of Disease**: Patients with overt skeletal manifestations of SHPT (e.g., osteitis fibrosa cystica, subperiosteal bone resorption on X-ray, or brown tumors) inherently have very high bone turnover and large calcium deficits, predisposing them to severe HBS. In contrast, those with mixed uremic osteodystrophy or adynamic bone (often seen in longstanding diabetes or with calcimimetic overuse) have lower turnover and thus lower HBS risk. A bone biopsy (though rarely done pre-PTX) showing high turnover and abundant osteoid would strongly predict HBS. Similarly, very low pre-op bone mineral density could indicate high turnover bone loss.
- **Surgical Factors**: The extent and abruptness of PTH reduction at surgery significantly influence HBS development. Total PTX without autotransplantation causes the most complete and immediate PTH withdrawal, and thus confers the highest HBS risk [130]. Subtotal PTX or total PTX with a small autograft (e.g., forearm implant) theoretically leaves behind some PTH source to mitigate post-op hypocalcemia [131]. Even so, if the remnant tissue is insufficient or non-functioning, HBS can still occur. Thus, cohorts receiving autotransplant have shown inconsistent results: either marginally lower rates of severe HBS compared to those with solely total PTX [132], or no effect at all in any type of HPT patient [11]. The size and weight of resected parathyroid glands may also correlate with risk—larger glands (or higher total gland weight) theoretically indicate a greater burden of hyperactive tissue, which in turn implies more profound skeletal PTH effects pre-op [11]. Even so, results are scarce and inconsistent. Notably, in primary HPT, glands >1.7 cm had higher HBS occurrence risk [133], whereas in SHPT, removal of very large hyperplastic glands likewise portends HBS [117]. Concomitant thyroidectomy has been noted as a risk factor in primary HPT (likely due to longer associated operative time) [11], but in SHPT patients this is less common.

Overall, patients exhibiting these risk factors should be counseled preoperatively about HBS and monitored closely after PTX. In high-risk individuals, preemptive measures have been advocated, such as intensified calcium/vitamin D loading in the days before and after surgery, or a planned autotransplant to retain partial PTH function [131]. While such strategies must be balanced against surgical curative goals, anticipation of HBS allows for prompt therapy and may reduce complications (e.g., arrhythmias, seizures due to hypocalcemia).

### 7.2. Emerging Predictive Models and Risk Scores

Beyond individual risk factors, recent efforts have shifted toward composite prediction tools for HBS. Traditionally, risk stratification was informal, relying more on clinical intuition, whereas nowadays, remarkably accurate and clinically practical, validated predictive models are steadily emerging. The NYU Langone Health team led by Ramesh et al. (2023) proposed a simple two-point risk score based on preoperative PTH and ALP levels [17]. In their cohort of dialysis patients with SHPT, HBS incidence was ~48%, yet the mean differences between HBS and non-HBS patients were striking: PTH levels differed by 2167.2 pg/mL (*p* < 0.001), phosphorus by 3.5 mg/dL (*p* < 0.001), and ALP by 344.2 U/L (*p* = 0.002). Stepwise regression identified ALP >150 U/L and markedly elevated PTH >1000 pg/mL as the key independent predictors [17]. Authors assigned one point for each criterion; a score of 2 points (both risk factors present) predicted HBS with 100% positive predictive value (all patients meeting both thresholds developed HBS) [17]. Even a score of 1 point carried a 93.8% positive predictive value, while a score of 0 points effectively ruled out HBS in their series [17]. This simple two-factor model demonstrated 96.8% overall accuracy (sensitivity 100% and specificity 94.1%) on internal validation, illustrating its potential clinical utility [17]. For instance, a dialysis patient with ALP twice the ULN and PTH > 1000 pg/mL would be flagged as high-risk (2 points), prompting preemptive calcium infusion and intensive monitoring.

In parallel, broader multivariate risk scores have been proposed using large datasets. Amjad et al. (2024) recently analyzed 17,074 PTX cases, performed between 2010 and 2021, from a national registry (US Renal Data System) to derive a weighted HBS risk index [134]. Five independent preoperative HBS predictors were identified on multivariable analysis: younger age, evidence of high bone-turnover (i.e., renal osteodystrophy), longer dialysis vintage, history of kidney transplant (reflecting long-standing SHPT), and higher comorbidity burden (Elixhauser score) [134]. Thereafter, based on these clinical factors, a scoring system was developed, with a total of 6 possible points (one point for each factor over threshold, except age < 48 years which add 2 points). Patients with 0 points had 8% HBS incidence, whereas those with the maximum 6 points had ~44% incidence [134]. Notably, while the positive predictive value of the score was modest (~20% for the highest-risk group), the negative predictive value was excellent—0 points effectively ruled out HBS in ~92% of cases [134]. This kind of model is useful for identifying low-risk patients who may not require extensive postoperative hospitalization and/or prolonged monitoring [134].

Another approach from China by Wang et al. (2020) used a more sophisticated nomogram, incorporating four independent HBS predictors (preoperative iPTH level, bone-specific ALP level, total weight of resected parathyroid glands, and lower preoperative corrected calcium), on a cohort of 114 patients with renal SHPT, undergoing total PTX with autotransplantation [117]. Their prospective data collection revealed an unexpectedly high 76.3% HBS occurrence rate, substantially exceeding Western populations [117]. Internal validation using bootstrapping demonstrated superior prediction performance compared to individual biomarkers alone [117].

Recently, Gao et al. (2024) refined their nomogram approach on a cohort of 75 maintenance hemodialysis patients, developing a prediction model of HBS risk after total PTX [135]. This model achieved exceptional performance with a C-index of 0.943 (95%CI: 0.892–0.994). The area under the curve for PTH alone was 0.873, while ALP reached 0.926. Optimal cutoff values were established for PTH (2433.1 pg/mL) and ALP (289.5 U/L), providing clinically actionable thresholds for risk stratification [135].

Conversely, machine learning applications show promise, with Boruta feature selection [17] and XGBoost algorithms [136] demonstrating superior performance compared to traditional logistic regression models. These advanced computational approaches enable integration of multiple variables, while accounting for complex interactions between biochemical markers and clinical factors [17,136].

Such nomograms and machine-learning models are still in early stages but show promise in individualized risk estimation. In essence, these tools synthesize the well-known predictors (extreme PTH elevation, high BTMs, low pre-op calcium, and younger patients with robust skeletal mass, etc.) into a more quantitative risk stratification. While no single model has been universally adopted yet, their emergence reflects a modern shift toward proactive risk management in SHPT. Patients identified as high-risk can be targeted for preventive interventions, e.g., initiating prophylactic calcium infusions guided by preoperative ALP levels [137], closer electrolyte monitoring, or delayed hospital discharge until calcium plateaus. Conversely, low-risk patients may be safely managed with standard postoperative care without needless intensive care unit (ICU) stays [134].

In clinical practice, integrating these risk predictors—possibly via an HBS risk calculator—can improve patient counseling and tailor postoperative care. As evidence grows, it may become standard to stratify all SHPT patients by HBS risk prior to PTX. This personalized approach aims to mitigate HBS-related morbidity and optimize the safety of curative surgery for SHPT.

## 8. Prevention and Management Strategies

Given the pathophysiological basis of HBS—a skeleton starved for minerals—the cornerstone of clinical management centers on anticipation (risk stratification, pre-optimization) and support (aggressive Ca^2+^/active vitamin D supplementation with close monitoring) [138]. Intravenous (IV) calcium gluconate is typically initiated in the operating room or recovery area for high-risk patients, with frequent dose titration based on serial ionized calcium measurements (e.g., every 6–8 h in the first day). High-dose active vitamin D analogs (e.g., calcitriol 2–4 μg/day IV or orally) are initiated early to maximize intestinal calcium absorption and accelerate bone mineralization rate. By upregulating osteoblast calcium-transporting ATPases and other enzymes, calcitriol helps drive the incorporation of calcium into deposited osteoid [139].

In fact, one retrospective study demonstrated that a “high-dose calcitriol protocol”—administering alfacalcidol ~12 µg/day (+ ample calcium supplementation) immediately after PTX, instead of the standard ~4 µg/day—significantly reduced the incidence of severe hypocalcemia and the need for IV calcium, compared to standard dosing [138]. In that study, the proportion of patients with critically low calcium (<1.5 mmol/L) was dramatically lower in the high-calcitriol group (e.g., 8.5% vs. 47% on post-op day 4), and only ~8% of those high-dose patients required rescue IV calcium, versus ~46% in the standard-dose group [138]. Consequently, early and ample vitamin D analog therapy perioperatively is recommended (barring contraindications) to help the bone mineralize faster and shorten HBS duration, i.e., fewer episodes of severe hypocalcemia and a shorter length of hospital stay [138]. Notably, earlier concerns that vitamin D repletion might exacerbate preoperative hypercalcemia have been allayed by clinical studies showing that vitamin D supplementation can safely lower PTH and bone turnover before surgery without causing significant calcium elevations [27].

Prophylactic bisphosphonates have also been explored as a strategy to blunt HBS, mainly in primary HPT (but conceptually relevant to SHPT as well). By inhibiting osteoclast-mediated bone resorption, pre-op bisphosphonates could theoretically reduce the post-PTX calcium influx into bone. Small studies in primary HPT support this idea: for example, Mayilvaganan et al. reported that none of the patients who received a pre-op zoledronic acid infusion developed HBS, versus 3 of 8 patients (38%) who did not receive bisphosphonate [140]. The bisphosphonate-treated group also had significantly lower post-op IV calcium requirements and shorter hospital stays than controls [140]. Similarly, earlier work by Lee et al. showed bisphosphonate pretreatment attenuated the drop in calcium after PTX in primary HPT patients [141].

In the setting of SHPT, there is some evidence that bisphosphonates can mitigate HBS severity, though data are limited to uncontrolled reports. Davenport et al. administered IV pamidronate ~24–48 h pre-PTX to a series of dialysis patients with refractory SHPT: this nearly abolished immediate severe hypocalcemia—only 2 of 27 bisphosphonate-treated patients required post-op calcium boluses, compared to all 10 patients who received standard care alone [142]. The pamidronate group maintained higher nadir calcium levels (median ~2.3 vs. 2.1 mmol/L) and had much lower intensive calcium supplementation needs [142]. Importantly, pretreated patients also had reduced monitoring demands and a shorter hospital stay (5.7 ± 2.9 vs. 9.2 ± 1.9 days) in that series [142]. However, these benefits must be weighed against potential downsides.

Bisphosphonates in ESRD can drive adynamic bone pathology or delay normal bone healing, as suggested by Davenport’s observation that bone density gains after surgery were slightly dampened in the pamidronate group [142]. Moreover, no randomized trials in CKD have proven the efficacy and safety of routine bisphosphonate use for HBS prevention, and current expert guidelines have not endorsed this practice. A recent systematic review found only scant, heterogeneous evidence for any preoperative intervention to reduce HBS risk, underscoring continued uncertainty in the field [137]. Given the availability of modern calcimimetics and high-dose calcitriol (which can be optimized without lasting skeletal effects), routine bisphosphonate prophylaxis is not recommended by KDIGO for SHPT [4]. It may be reserved for select extreme cases (e.g., very high ALP and bone turnover) on an individualized basis, after multidisciplinary review. In summary, while bisphosphonate pretreatment appears beneficial in some small studies, the lack of robust CKD-specific data and the risk of adynamic bone mean that broad use cannot be advised at this time.

Another emerging therapy discussed in this context is Denosumab (a RANKL inhibitor). In theory, a preoperative dose of denosumab could sharply curtail osteoclast activity and bone resorption, thereby reducing the post-PTX “hungry bone” effect. However, this approach remains speculative, being thus far purely experimental and untested in practice. There is significant concern that denosumab itself can precipitate hypocalcemia in CKD patients—in fact, the FDA recently issued a boxed warning that denosumab (Prolia) markedly increases the risk of severe, symptomatic hypocalcemia in patients with advanced CKD, especially those on dialysis [143]. Until more data are available, denosumab is not an established strategy for HBS prevention, and its use would be contraindicated or approached with extreme caution in this setting. To sum up, Denosumab (anti-RANKL) remains experimental here; given its hypocalcemia risk in advanced CKD, we do not recommend routine use for HBS prevention outside of exceptional, individualized scenarios.

From a surgical standpoint, if a patient is deemed extremely high-risk for HBS, some surgeons have considered altering the operative strategy to mitigate the abrupt cessation of PTH. Options include performing a subtotal PTX (leaving a small remnant of parathyroid tissue in situ) or autotransplanting a piece of resected parathyroid into the forearm or neck, with the aim of retaining partial PTH function to ease the post-op decline [131]. These approaches allow a more gradual reduction in PTH levels, theoretically tempering the bone’s mineral uptake. However, such strategies must be weighed against the risk of persistent or recurrent HPT. In general, the indications for surgery (refractory severe SHPT +/− complications) demand maximal PTH reduction, so most surgeons proceed with a total PTX (often without forearm autograft) and manage the ensuing HBS medically, rather than compromise the curative surgical goal. There has been some interest in *staged* PTX (removing two glands first, then the remaining glands in a second operation) to spread out the PTH drop [144], but this approach is rarely used due to the need for multiple surgeries and unclear benefit.

Intensive postoperative management protocols are the mainstay for HBS. Many centers initiate calcitriol or alfacalcidol loading a few days before surgery—for example, starting 2–4 µg/day of calcitriol, 1–2 days pre-op—to ensure vitamin D stores are replete and possibly to modestly suppress PTH secretion before the big drop [145,146]. It is prudent to correct nutritional vitamin D deficiency (25-hydroxyvitamin D > 30 ng/mL) prior to PTX, as severe deficiency could exacerbate post-op hypocalcemia [147]. Postoperatively, high-risk patients are often managed in an ICU or high-dependency unit for continuous calcium infusion titration and cardiac monitoring [27]. Frequent laboratory monitoring is essential: serum calcium (total and ionized), magnesium, and phosphate are checked often (typically twice daily or at least once a day for the first 1–2 weeks) [148]. Aggressive supplementation is continued until the nadir phase of HBS passes. Patient education is also critical—patients should be counseled on the symptoms of hypocalcemia (perioral numbness, muscle cramps, tingling, etc.) and the importance of strict adherence to supplementation medications and follow-up lab appointments after discharge.

Despite best efforts, HBS may still occur [149]; thus, post-PTX management is often prolonged. Typical therapy for established HBS includes high-dose oral calcium (e.g., 3–4 g of elemental calcium daily, divided into multiple doses—usually every 6 h), and calcitriol (often up to ~4 µg/day), continued upon discharge, with a gradual taper over several weeks as the bone remineralizes [15]. Serum ALP and calcium trends guide the tapering—as ALP levels fall and stabilize (indicating slowing bone formation) and serum calcium normalizes, supplements can be slowly reduced. These intensive supplementation doses are generally safe and necessary in this context, given the extraordinary calcium demands; for example, daily elemental calcium intakes of 6–12 g and calcitriol 2–4 µg have been used previously, in very severe HBS cases [15]. The patient’s cardiac rhythm should be monitored during periods of severe hypocalcemia. In cases of life-threatening hypocalcemia (e.g., arrhythmias, seizures), calcium boluses and even continuous telemetry in an ICU setting are indicated until stabilization.

Phosphate management is another consideration: profound hypophosphatemia may accompany HBS (as phosphate is also avidly taken up by bone). If phosphate levels fall very low, cautious supplementation is indicated—either increasing dietary phosphate intake or giving IV phosphate in small doses—while avoiding overshooting (since renal failure patients cannot excrete phosphate easily) [150]. In practice, dialysis prescriptions and diet usually supply enough phosphorus over time, but levels should be monitored [151]. Magnesium repletion is also important. Hypomagnesemia, common in dialysis patients, reduces PTH secretion and end-organ PTH responsiveness, compounding hypocalcemia [152]. Thus, magnesium should be maintained in the mid-normal range (e.g., via oral magnesium oxide or IV magnesium sulfate) to help stabilize calcium levels [153]. Additionally, if metabolic alkalosis is present (for instance, from aggressive bicarbonate dialysis or citrate), it should be corrected, as alkalosis increases calcium binding to albumin and can worsen symptoms at a given calcium level [152].

Close collaboration between surgeons, nephrologists, and intensive care is often necessary during the acute management of HBS. Dialysis adjustments can help as well (e.g., using a higher calcium bath in hemodialysis treatments post-PTX to aid calcium balance). Each patient’s course is variable; thus, frequent lab monitoring (often multiple times daily in the first week) guides therapy. As days pass and bone hunger gradually abates, calcium infusion rates can be tapered and eventually switched to oral only. Patients are usually maintained on oral calcium and calcitriol for many weeks or months after discharge, with outpatient monitoring to ensure calcium remains stable and to prevent a relapse of hypocalcemia. As HBS begins to resolve (typically evident by normalization of ALP and stability of calcium on minimal supplementation), therapy can be weaned. Nonetheless, a subset of patients, especially those who underwent total PTX, may transition directly from HBS into a state of permanent hypoparathyroidism where lifelong calcium/vitamin D support is needed. Distinguishing prolonged HBS from true hypoparathyroidism can be challenging; one criterion is the recovery of PTH levels—if PTH remains undetectable long-term and calcium cannot be maintained without supplements beyond 1 year, permanent hypoparathyroidism is likely.

Paradoxically, the main beneficial consequence of HBS is a robust improvement in bone density. The massive influx of minerals into bone that causes transient hypocalcemia also leads to rapid gains in bone mineral content once the pre-deposited osteoid reserve pool fully mineralizes. Patients who endure an episode of severe HBS often show markedly increased bone mineral density at 6–12 months post-PTX and experience relief of prior bone pain. Some observational studies even suggest that experiencing HBS (versus not) correlates with better longer-term individual outcomes, perhaps because it signifies a more complete correction of the preexisting high-turnover bone disorder. For example, one recent analysis found that dialysis patients who developed HBS had more pronounced improvements in blood pressure, anemia, and nutritional status after PTX, compared to those without HBS [150].

Nonetheless, this “rebound osteogenesis” should not be misinterpreted as an argument for HBS, yet it highlights that once patients are guided through the acute phase safely, they tend to reap substantial benefits from the surgery—improved quality of life, less skeletal pain, possibly fewer fractures, and reversal of many features of renal osteodystrophy [150]. The goal, therefore, is to manage HBS proactively so that patients can reach this recovery phase without undue morbidity. In essence, the “hungry bones” will eventually be sated; with meticulous supportive care, we can satisfy their mineral demands while protecting the patient. The occurrence of HBS can then be viewed as a sign of successful elimination of severe hyperparathyroid bone state—a difficult perioperative course that may ultimately yield healthier bones.

## 9. Conclusions

HBS remains a challenging, yet predictable complication following PTX for SHPT in ESRD/dialysis patients. It represents the culmination of CKD-induced high-turnover bone disease: the skeletal metabolism, suddenly liberated from chronic excess PTH signaling postoperatively, dramatically shifts from net mineral efflux to net influx, voraciously incorporating minerals (mainly calcium) into the abundant pre-deposited osteoid reserve pool, resulting in prolonged, profound electrolyte disturbances, i.e., aggressive refractory hypocalcemia. In this review, we explored and expanded upon the current understanding of HBS pathogenesis, emphasizing the molecular and cellular events—the suppression of osteoclast activity (via RANKL/OPG downregulation), the persistence of osteoblast-driven osteogenesis through Wnt/β-catenin signaling (aided by transiently low sclerostin levels), osteocytic network dysregulation, and CaSR-mediated mineral uptake potentiation responses—that together create an extreme calcium demand in bone post-PTX. Herein, we highlighted how preoperative factors, such as elevated BTMs, high PTH, and large cumulative osteoid stores, set the stage for severe HBS. Thereafter, we analyzed the temporal succession of HBS phases during its natural evolution (immediate, acute, subacute nadir, recovery), along with their clinical implications.

For the practicing clinician, key management priorities include (1) preoperative risk stratification using simple scoring systems, (2) aggressive perioperative calcium and vitamin D supplementation in high-risk patients, (3) frequent monitoring during the critical nadir phase (days 2–14), and (4) gradual tapering guided by normalizing ALP levels rather than calcium alone. Recognition that 10–15% of patients experience protracted HBS, requiring >1 year of supplementation, is essential for appropriate counseling and follow-up planning.

From a practical standpoint, recognizing which patients are at highest risk of severe HBS (for example, a young dialysis patient, with very high ALP/PTH levels, undergoing total PTX) allows for preoperative personalized prophylactic measures to be taken, respectively, for proactive mitigation early on. During the critical postoperative period, meticulous management with aggressive calcium supplementation (often IV) and active vitamin D analogs, coupled with frequent monitoring of electrolytes, is essential and can be lifesaving. While the early postoperative course can be difficult, it is important to remember that HBS is, in effect, a consequence of successfully eliminating an extreme hyperparathyroid state. Most patients, even those with profound HBS, will eventually stabilize over weeks to months as their bone remineralization completes. Over the long term, they typically enjoy significant improvements: increased bone mineral density, reduction in bone pain, and other benefits related to resolving the CKD–mineral and bone disorder. In this sense, the occurrence of HBS can be viewed as the “price paid” for restoring bone metabolism to normal—a transient period of imbalance on the path to healthier bones.

Future directions in HBS research and care are aimed at refining risk stratification and exploring adjunct therapies to lessen severity. Incorporating genetic markers (such as polymorphisms in the VDR or CaSR) into risk models may improve our current predictive ability for HBS occurrence and severity. Investigating novel perioperative treatments—for instance, using anabolic agents or anti-resorptive drugs in the immediate pre or postoperative period to blunt the extreme “bone hunger” (with caution given CKD patients’ unique physiology)—holds promise for preventing the most severe manifestations. Another practical question meriting investigation is the management of calcimimetic therapy around surgery: whether continuing calcimimetics up to the day of PTX versus withholding them affects the incidence or severity of HBS.

In conclusion, HBS exemplifies the remarkable plasticity of the adult skeleton and the delicate balance of mineral homeostasis. By understanding the pathogenesis of HBS in the context of SHPT, clinicians and surgeons can better anticipate its occurrence and implement targeted strategies to prevent and manage it. The bones may indeed be “hungry” post-PTX, but with vigilant supportive care we can sate their appetite efficiently, while safeguarding our patients’ well-being, thus guiding them through the critical postoperative period, towards a state of improved health and restored bone equilibrium.

## Figures and Tables

**Table 1:** Clinical phases of hungry bone syndrome after parathyroidectomy in secondary hyperparathyroidism with associated clinical/biochemical features and summarized management considerations.

Phase (Post-PTX)	Time Frame	Key Biochemical Changes	Clinical Features & Management Considerations
Immediate	0–6 h	PTH drops >90% within minutes; Ca^2+^ initially stable (due to buffers).	Typically, asymptomatic [121,122]. Initiate calcium prophylaxis (e.g., IV calcium), especially if pre-op PTH was high [12]. Monitor Ca^2+^ q6h [27].
Early Acute	12–72 h	Ca^2+^ starts to fall (notably by 18–24 h); PO_4_^3−^ rapidly falls by 24–48 h; PTH at nadir.	Subtle symptoms (perioral tingling, distal paresthesias, muscle cramps) may ensue. Positive Chvostek/Trousseau may be elicited [27]. Intensive Ca^2+^ monitoring (q6–8h) required; adjust IV/oral Ca^2+^ upwards as needed [27]. Ensure serum Mg^2+^ is normal [14]. Calcitriol analogues should be initiated [27].
Subacute	Day 3–14	Ca^2+^ reaches nadir (often day 5–7); ALP peaks (2–3× pre-op); PO_4_^3−^ often <2 mg/dL; Mg^2+^ low as well. BTMs (CTX, TRAP5b) at nadir.	Severe hypocalcemia symptoms may appear: tetany, seizures, bronchospasm, laryngospasm, arrhythmias. Cardiac monitoring mandatory [15,16]. Highest IV Ca^2+^ requirements (oftentimes >10 g calcium gluconate/24 h) [12]. PO_4_^3−^ supplementation if <1.5 mg/dL [12]; administer IV Mg^2+^ as needed [14]. Calcitriol doses should be maximized [12].
Recovery	2 weeks—12 months	Ca^2+^ gradually normalizes (weeks); ALP declines toward normal (months); PTH remains low (if total PTX) or low-normal (if subtotal/PTX+autograft).	Symptoms resolve. Taper IV Ca^2+^, then oral Ca^2+^ over weeks-months. High calcitriol dose continued until ALP normalizes and there is no hypocalcemia on minimal Ca^2+^ supplementation [15]. In ~10–15% of patients, oral Ca^2+^/vitamin D needed >1 year (“protracted HBS”) [16]. Endocrine follow-up for permanent hypoparathyroidism (if persistent) [27].

Abbreviations: Ca^2+^ = calcium; PTH = parathyroid hormone; PO_4_^3−^ = phosphate; ALP = alkaline phosphatase; BTM(s) = bone turnover marker(s); CTX = C-terminal telopeptide of type I collagen; TRAP-5b = tartrate-resistant acid phosphatase 5b; Mg^2+^ = magnesium; IV = intravenous; q6h = every 6 h (similarly, q6–8h = every 6 to 8 h); PTX = parathyroidectomy.

**Table 2:** Key preoperative risk factor studies for hungry bone syndrome after parathyroidectomy in secondary hyperparathyroidism.

Study (Year)	Patient Population (n, Details)	HBS Incidence (Definition)	Significant Preoperative Risk Factors for HBS
Ho et al., 2017 [30]	62 dialysis patients; tPTX(-)AT; 10-year single-center cohort.	27.4% (corrected Ca^2+^ ≤ 2.1 mmol/L, lasting for ≥4 days, within 1st month post-PTX)	Younger age, higher body weight, higher ALP and lower Ca^2+^ predicted HBS in MLR analysis. iPTH was elevated in HBS vs. non-HBS, but not an independent predictor (when adjusted for ALP).
Ge et al., 2019 [114]	115 dialysis patients; tPTX(+)AT; 2.5-year single-center cohort.	87.8% (total Ca^2+^ ≤ 2.1 mmol/L and/or hypocalcemia for ≥4 days post-PTX)	Higher ALP and lower Ca^2+^ predicted HBS in MLR analysis. Younger age, higher ALP and higher iPTH positively correlated with HBS severity. Specific bone metabolism dynamics were revealed (post-PTX: iPTH, CT, CTX and TRACP5b rapidly decreased, while OC and ALP increased more slowly).
Kritmetapak et al., 2020 [18]	130 dialysis patients; PTX technique at surgeons discretion; 6-year single-center cohort.	82.3% (Ca^2+^ nadir <8.4 mg/dL within the first 3 days post-PTX and/or requiring IV Ca^2+^ for symptoms)	PTH >1000 pg/mL, ALP >420 U/L, age ≤45 years and absence of hypercalcemia (corrected Ca^2+^ <10.2 mg/dL) were significantly associated with HBS in MLR analysis.
Phimphilai et al., 2022 [116]	179 dialysis patients; mostly tPTX(+)AT (79.3%); 22- year single-center cohort.	82.1% (corrected Ca^2+^ <8.5 mg/dL for >3 days post-PTX)	Longer dialysis vintage (≥5 years), higher PO_4_^3−^ (≥5 mg/dL); higher ALP (≥387 U/L) and mean difference between iPTH pre- and post-PTX (>97%) were independent risk factors for hypocalcemia in MLR analysis.
Gao et al., 2022 [129]	2990 ESRD patients with rSHPT (13 studies, from 2013–2021); variable PTX techniques (including ablation).	Not applicable (Meta-analysis of risk factors for post-PTX hypocalcemia)	Lower Ca^2+^, higher ALP and higher iPTH were associated with post-PTX hypocalcemia (i.e., Ca^2+^ <8.4 mg/dL within first 3 days post-PTX). Age was not significant.
Mehta et al., 2024 [11]	2598 patients undergoing PTX for primary, secondary and tertiary HPT (18 studies, from 2006–2021); variable PTX techniques.	43.6% for SHPT cohort (Systematic review of risk HBS factors)	Younger age, larger glands, previous dialysis, longer dialysis vintage, Ca^2+^ close to normal, higher iPTH (i.e., >90% drop in PTH post-PTX), higher ALP, higher OC, lower albumin, and higher TRACP5b were HBS predictors in the SHPT cohort

Abbreviations: ALP = alkaline phosphatase; BTMs = bone turnover markers; Ca^2+^ = calcium; HBS = hungry bone syndrome; CT = calcitonin; CTX = C-terminal telopeptide; IV = intravenous; MLR = multiple logistic regression; OC = osteocalcin; PO_4_^3−^ = phosphate; (i)PTH = (intact) parathyroid hormone; PTX = parathyroidectomy; tPTX(+/−)AT = total PTX (with/without) autotransplantation; rSHPT = refractory secondary hyperparathyroidism; TRACP5b = tartrate-resistant acid phosphatase 5b.

## Data Availability

Data available on request.

## Abbreviations

The following abbreviations are used in this manuscript:

ALP	Alkaline phosphatase
BMU	Basic multicellular unit (anatomic bone remodeling unit)
BTMs	Bone turnover markers
CaSR	Calcium-sensing receptor
CKD	Chronic kidney disease
cAMP	Cyclic adenosine monophosphate
CREB	cAMP-response element binding protein
C-PTHR	C-terminal parathyroid hormone receptor
CTX	C-terminal telopeptide of type I collagen (bone resorption marker)
CYP24A1	25-hydroxyvitamin D-24-hydroxylase
CYP27B1	25-hydroxyvitamin D-1α-hydroxylase
ESRD	End-stage renal disease
FGF-23	Fibroblast growth factor-23
GFR	Glomerular filtration rate
H&E	Hematoxylin and eosin (histological stain)
HBS	Hungry bone syndrome
HPT	Hyperparathyroidism
iPTH	Intact parathyroid hormone (full-length 1–84 PTH)
MCP-1	Monocyte chemoattractant protein-1
MEF2	Myocyte enhancer factor 2
MLR	Multivariate logistic regression
NaPi-2a	Sodium-phosphate co-transporter type 2a
NaPi-2c	Sodium-phosphate co-transporter type 2c
NTX	N-terminal telopeptide of type I collagen (bone resorption marker)
OC	Osteocalcin (osteogenesis marker)
OPG	Osteoprotegerin
PKA	Protein kinase A
PKC	Protein kinase C
PTH	Parathyroid hormone
PTH1R	Type 1 parathyroid hormone receptor (classical PTH receptor)
PTX	Parathyroidectomy
P1NP	Procollagen type I N-terminal propeptide
RANK	Receptor activator of nuclear factor-κB (osteoclast precursor receptor)
RANKL	Receptor activator of nuclear factor-κB ligand
SHPT	Secondary hyperparathyroidism
TRACP5b	Tartrate-resistant acid phosphatase 5b (resorption marker)
TRPV5	Transient receptor potential vanilloid 5 (renal calcium channel)
TRPV6	Transient receptor potential vanilloid 6 (intestinal calcium channel)
ULN	Upper limit of normal (reference range)
VDR	Vitamin D receptor
